# Gonococcal Infective Endocarditis Returns

**DOI:** 10.7759/cureus.17955

**Published:** 2021-09-14

**Authors:** Mina Said, Ekta Tirthani

**Affiliations:** 1 Internal Medicine, Rochester Regional Health, Rochester, USA

**Keywords:** gonococci, infective endocarditis, disseminated gonococcal infection, septic emboli, sexually transmitted disease

## Abstract

Disseminated gonococcal infection occurs in 0.5%-3% of gonorrhea cases, usually in the form of either a triad of arthralgia, tenosynovitis, and skin lesions or purulent arthritis. Other rare complications include gonococcal infective endocarditis that occurs in 1%-2% of cases with 99 cases reported in the literature since 1938. Our case presents an additional rare case of aortic valve gonococcal endocarditis requiring surgical intervention and a prolonged antibiotic course, despite the absence of genitourinary symptoms or mucosal evidence of infection. This case was found to have sepsis and gonococcal endocarditis, which was clearly confirmed with positive blood cultures and aortic valve vegetation. It was further complicated by the evidence of splenic embolization and severe aortic regurgitation requiring surgical valve replacement and debridement of an annular perivalvular abscess. A high degree of suspicion is needed to early diagnose these unusual cases of gonococcal endocarditis, especially in sexually active individuals, for its known virulence causing valve destruction and high mortality. Our case represents a valuable addition to the reported cases of this diagnosis and is complemented by a short literature review.

## Introduction

*Neisseria gonorrhea* was discovered in 1879 [1]. It is currently the second most common reportable sexually transmitted disease (STD) after chlamydia [2], with an annual incidence of 700,000 new cases in the United States [3] and more than 86 million cases worldwide [3,4], posing new expansion and resistance challenges to public health [5]. In recent years, it has gained more attention since the rate of multidrug-resistant gonorrhea and asymptomatic cases are rising [6], with a reported tendency to facilitate HIV transmission [3].

Therefore, the Center for Disease Control and Prevention (CDC) recommends gonococcal screening for females below the age of 25 years, homosexual males, and HIV patients at risk annually [7]. Gonococcal infections are known for causing possible disseminated complications in up to 3% of cases. Gonococcal endocarditis is a rare complication of gonorrhea and also a rare cause among infective endocarditis-causing pathogens. It is a highly important diagnosis, given the occasionally difficult lab identification, the high degree of suspicion needed for diagnosis, and the significant proportion of associated fatal complications. Our case represents a valuable addition to the literature, and a comparison is provided with other cases of the post-antibiotic era [5,8-13].

## Case presentation

A 26-year-old Caucasian female with a past medical history of asthma, hypertension, polycystic ovary syndrome, and obesity presented to the hospital with a two-week history of multiple progressive complaints including intermittent fevers with chills, headaches, dry cough, shortness of breath on exertion, chest discomfort that is exacerbated by coughing, nausea, vomiting, and decreased appetite. She denied any sputum production, hemoptysis, wheezing, leg swelling, change in bowel habits, skin lesions, arthralgia, or genital symptoms. She had a past history of smoking, with occasional alcohol use but no drugs. She was sexually active with males, denied risky sexual behaviors or previous STDs, and used progesterone implants for contraception. Family history was non-significant. Her vital signs showed blood pressure of 151/72 mmHg, tachycardia at 131 bpm, respiratory rate of 36/min, fever of 38.6°C, and oxygen saturation of 99% on room air.

On physical exam, she had tenderness over the sternum area, the epigastrium, and left upper abdominal quadrant. Heart sounds suggested a faint aortic diastolic murmur. She was noted to become rapidly dyspneic after moving for short distances. There were no skin lesions, joint tenderness, or effusions. Her workup showed white blood cells of 10.4 x 10^9^/L, hemoglobin of 9.0 g/dl, and platelets of 401 x 10^9^/L; urinalysis showed incidental trichomoniasis, unremarkable basic metabolic panel, procalcitonin of 0.93 ng/ml, lactic acid of 0.8 mmol/L, troponin of 0.07 ng/ml, brain natriuretic peptide (BNP) of 59 pg/ml, d-dimer of 1343 ng/ml, sedimentation rate of 72 mm/hr, and C-reactive protein (CRP) of 130 mg/L. Severe acute respiratory syndrome coronavirus 2 (SARS-CoV-2) and atypical/viral testing were negative. Chest x-ray was normal, and chest CT with contrast showed small non-specific zones of airspace disease but no pulmonary emboli or upper abdominal lesions. She was subsequently admitted for sepsis, with two sets of blood cultures drawn, and started on ceftriaxone and doxycycline for sepsis of possibly respiratory origin as well as metronidazole for trichomoniasis.

**Figure 1:**
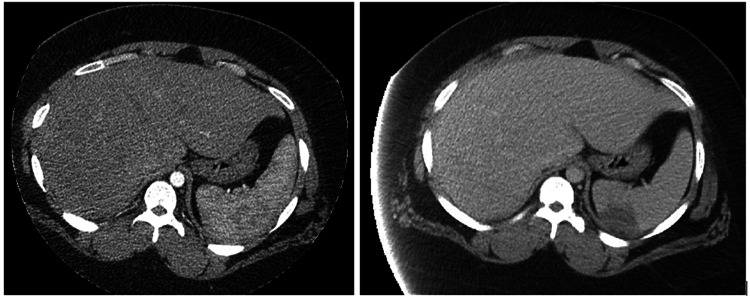
Abdominal CT showing a new 4.3-cm splenic infarct compared to the initial chest CT lower films of the previous day

The following day, she remained febrile and developed generalized abdominal pain, emesis, and diarrhea. An abdominal CT was done, which showed a new 4.3-cm splenic infarct compared to the initial chest CT films (Figure 1). Her blood cultures were first falsely reported as growing gram-positive cocci in clusters from the two aerobic bottles; then on day 3, they were identified as *Neisseria gonorrhea*, which was reported to the local department of health. The patient underwent a transthoracic echocardiogram that showed echogenic vegetation on the aortic valve with a moderate-to-severe aortic regurgitation. Subsequently, a transesophageal echocardiogram was done confirming a 1.5-cm vegetation on the left coronary cusp of the aortic valve with severe aortic regurgitation and no abscess collections. Gonococcal infective endocarditis with splenic embolization was confirmed. Infectious diseases and cardiothoracic teams were consulted.

On day 4, tests for syphilis antibodies, hepatitis B and C screening, and chlamydia were all negative. She underwent recto-vaginal and pharyngeal swabs for gonococcal nucleic acid amplification testing and cultures, both of which were surprisingly negative. Doxycycline and metronidazole were stopped. On day 5, the susceptibilities of the cultures showed resistance to ciprofloxacin and tetracycline but good sensitivity to ceftriaxone, with negative repeat blood cultures. Ceftriaxone dose was increased to 3 g daily due to her body weight of more than 150 kg. On day 6, she underwent mechanical aortic valve replacement with intraoperative findings of a 3-mm annular abscess. Gentamicin was added for synergy, but follow-up susceptibilities showed resistance to it, and so it was stopped. She remained in the cardiothoracic intensive care unit with gradual improvement of symptoms till the chest tubes were removed. Her CH50 was tested to rule out complement deficiency and was normal. The valve tissue sample, vegetation, and annular abscess material were sent for Gram staining, which showed only rare white blood cells with no organisms, and also for aerobic and anaerobic cultures, which did not grow any bacteria. She was started on warfarin and discharged on day 10 with a plan for six weeks of 3 g IV ceftriaxone monitored with the infectious diseases clinic. Her course was complicated with one episode of chest pain with the cardiac workup, CT chest and a repeat transthoracic echocardiogram were all unremarkable, and the antibiotics course was completed successfully. She remains medically stable at a 90-day follow-up.

## Discussion

Disseminated gonococcal infection (DGI) occurs in 0.5%-3% of gonorrhea cases [14], mostly secondary to local mucosal inflammatory tissue damage leading to protected transmission of *Neisseria gonorrhea* inside the neutrophils into the blood [3]. However, blood cultures are negative in 50% of DGI cases [15], which suggests that immune response might be responsible for the DGI symptoms instead. DGI usually presents in the form of either a triad of arthralgia, tenosynovitis, and skin lesions, or a more localized purulent arthritis form, or other rare complications like perihepatitis [16], osteomyelitis [16], vasculitis [17], and endocarditis [11].

Gonococcal infective endocarditis is estimated to have caused around 11%-26% of all infective endocarditis in the pre-antibiotic era, with 120 cases reported in the literature from 1895 to 1938 [1]. Currently, it occurs in 1%-2% of gonorrhea cases only, with a total of 99 cases reported in the antibiotic era till now. It is noteworthy that this incidence is directly related to the availability of the appropriate imaging and laboratory modalities as well. In terms of demographics, gonorrhea occurs in the United States most commonly in a younger age group ranging from 15- to 24-years old, of African-American ethnicity, with a male predominance, and at the southern US border [9,15,18]. Gonococcal endocarditis roughly follows the same pattern with a mean age of 28.8 years, and 57% of cases are in males [8,9]. Our case was relatively in the same age cohort but differed in being for females of Caucasian ethnicity and at the northeastern US region.

Gonococcal endocarditis usually presents subacutely in around two to four weeks, with generalized fatigue, fevers that can be double quotidian (two fever spikes per day), chills, jaundice in cases of liver involvement, preceding arthritis, petechial rash, renal dysfunction, and new cardiac murmurs [8,9]. Bundle branch block on electrocardiogram is sometimes noted [19]. Preceding mucosal symptoms are uncommon, with two-thirds having no genitourinary complaints at all, which probably drives clinicians away from suspecting this diagnosis in sexually active individuals and also predisposes these patients to dissemination due to untreated subclinical infections [15,20]. Our case did present with a subacute onset, fevers (not double quotidian pattern though), and chills but had none of the other aforementioned typical findings. In addition, her main presenting symptoms are considered uncommon for such a diagnosis compared to other previously reported cases. For example, dyspnea was reported in about 27% of a previous case series, and systemic embolization was identified in only 15.7% [8,21].

Gonococcal cardiac involvement is known to be aggressive with a tendency to cause rapid valve destruction and large vegetations despite appropriate antibiotic treatment [22]. This accounts for a 19%-23% mortality rate and more than half of the cases requiring urgent valve surgery [1]. It most commonly involves the aortic valve (50%), as in our case, followed by the mitral valve (24%), then the tricuspid valve (6%) [8]. A ring or perivalvular abscess has been reported in some cases. Diagnosis is usually made through gonococcal isolation on blood cultures. Other confirmation tests in culture-negative cases include genital and pharyngeal mucosal gonococcal swabs, the new matrix-assisted laser desorption/ionization time-of-flight mass spectrometry (MALDI-TOF MS), or rarely valve tissue nucleic acid amplification testing [20]. Valve cultures are usually negative [9]. Our case had positive blood cultures and valvular vegetations but negative mucosal swab testing and valve tissue culturing. The falsely reported positive Gram stain was also previously reported in some cases in the literature [19].

As per the CDC Sexually Transmitted Infections (STI) Treatment Guidelines, the recommended treatment of gonococcal endocarditis is parenteral ceftriaxone for at least four weeks and one dose of oral azithromycin [15]. In the past, sulfonamides, penicillins, aminoglycosides, fluoroquinolones, and tetracyclines were used [4]. Over the years, *N. gonorrhea* has shown a concerning ability to develop resistance to multiple antibiotic agents, sometimes even to first-line treatment options like the third-generation cephalosporins recently [3,15]. Per the new CDC guideline updates, apart from increasing the dose of ceftriaxone for *N. gonorrhea*, sensitivity testing should be regularly performed for optimization of treatment and surveillance for resistance patterns [13]. Our case was indeed tested for sensitivities and found to be resistant to ciprofloxacin and tetracycline as mentioned. It is also essential to test and treat other STDs and sexual partners. Efforts are ongoing to develop new antibiotics like zoliflodacin, gepotidacin, and solithromycin [2] as well as a preventative vaccine for such a critical infection [5].

We have included citations of some previously reported cases since 1938 to our report for future reference of this interesting topic [6,8-12,23-45].

## Conclusions

Gonorrhea can present in different disseminated forms, usually without preceding genitourinary symptoms. Our case represents a valuable additional case of gonococcal infective endocarditis to the available literature of the antibiotic era, with the previous cases cited for the reader's reference per our review. It highlights the high degree of suspicion needed to make this unusual and potentially fatal diagnosis in sexually active individuals. It also presents some unusual features, including the initial presentation with heart failure symptoms like dyspnea on exertion as well as embolization phenomena as splenic infarcts. Valve replacement surgery and proper antibiotic course per current guidelines were proven to treat this patient successfully.
